# Organic Fusion of Molecular Simulation and Wet-Lab Validation: A Promising High-Throughput Strategy for Screening Bioactive Food Peptides

**DOI:** 10.3390/foods14162890

**Published:** 2025-08-20

**Authors:** Dongyin Liu, Yuan Xu, Xin Zhang, Fawen Yin, Jun Cao, Zhongyuan Liu, Dayong Zhou, Aiguo Feng, Chuan Li

**Affiliations:** 1 Hainan International Joint Research Center for Innovative Utilization of Tropical Seafood Resources, School of Food Science and Engineering, Hainan University, Haikou 570228, China; 2 School of Food Science and Technology, Dalian Polytechnic University, Dalian 116034, China

**Keywords:** molecular simulation, bioactive peptide, food protein, receptor protein, non-thermal technology, artificial intelligence

## Abstract

Peptides derived from protein sources in food exhibit a diverse array of biological activities. The screening, preparation, and functional investigation of bioactive peptides have become a focal area of research. This review summarizes the status of peptide activity mining, including the latest research progress in protein sources, peptide functions, and processing conditions. It critically evaluates the limitations of current bioactive peptide screening methods, including the drawbacks of traditional methods and molecular simulations. The potential of using molecular simulation for the virtual screening of potentially bioactive peptides is summarized. This includes virtual enzymatic digestion, molecular docking, simulation of non-thermal processing technologies, and the construction of organelle/cell models. The driving role of artificial intelligence in molecular simulation is also discussed. In addition, the structural information, mechanism, and structural analysis technique of action of the popular target proteins of foodborne bioactive peptides are summarized to provide a better reference for virtual-reality combinations.

## 1. Introduction

To date, proteins derived from animals and plants have been extensively studied as sources of bioactive peptides. These bioactive peptides benefit human health by influencing key bodily systems such as the cardiovascular, nervous, digestive, and immune systems. Existing reviews on aquatic foods-derived bioactive peptides have focused primarily on their functional activities, such as antioxidant, antihypertensive, antibacterial, anxiolytic, immunomodulating, anticancer, hyperuricemia-alleviating (or uric acid-lowering), and skin-improving properties [1]. However, they often neglect the latest trends, including high-throughput rapid screening, structure–activity relationship-guided precise design, and the development of bifunctional or multifunctional peptides. With the extensive exploration of peptide activities, the key protein receptors through which peptides exert their effects have been thoroughly analyzed to provide molecular evidence for the mechanisms of peptide activity. Against the backdrop of the rapid development of computer technology, the clarification of key information such as amino acid composition, three-dimensional structure, and active pockets has given rise to a novel method of screening bioactive peptides—virtual screening under molecular simulation.

Molecular simulation techniques rapidly analyze the affinity or interaction forces between peptides and receptor proteins. Compared with traditional experiments that rely on cell culture or animal validation, virtual screening offers the advantages of being fast and low-cost [1]. However, virtual screening has issues with false positives and false negatives. This is because the interactions between peptides and protein receptors are considered under ideal conditions without considering factors such as molecular crowding [2]. Despite these limitations, virtual screening remains a valuable tool for high-throughput screening of targeted bioactive substances. Moreover, virtual enzymatic digestion based on computer technology has been used to search for potential bioactive peptides [2]; and virtual organelle simulation has provided a new perspective for studying the interactions between peptides and receptor proteins [3].

Many reviews have been published on the applications of molecular simulation in food science technology. Ref. [4] reviewed the interaction between muscle proteins and exogenous additives by molecular docking and molecular dynamic simulation, which may be valuable research methods for improving the qualities of muscle foods. More recently, the principles of molecular docking, molecular dynamics simulation, and the molecular interaction mechanisms between food proteins and ligands from food processing have been reviewed [5]. Chang et al. [6] summarize the advantages and future of artificial intelligence (AI) techniques in screening foodborne bioactive peptides and emphasize that a general deep learning framework with multi-scale chemical spatial features can be used to predict peptide-target dynamic interactions. For research on AMPs, where the role of physicochemical features is utilized extensively and summarized numerous times, see, for example, Torres et al. [7]. However, the acquisition of peptides, activity screening, simulation of mechanisms of action, and the receptor proteins of peptide bioactivity based on computer simulation have not been well summarized. The role of AI in promoting molecular simulation technology also needs to be further summarized.

This review summarizes the distribution of bioactive peptides in food proteins and focuses on the advantages and future of using computer technology for peptide preparation, activity identification, and mechanism of action research. This review provides ideas and summaries for the high-throughput screening of bioactive peptides. Finally, it is suggested that combining molecular simulation and traditional wet-lab experiments is a more promising method for mining bioactive peptides.

## 2. Research Progress

**Figure 1:**
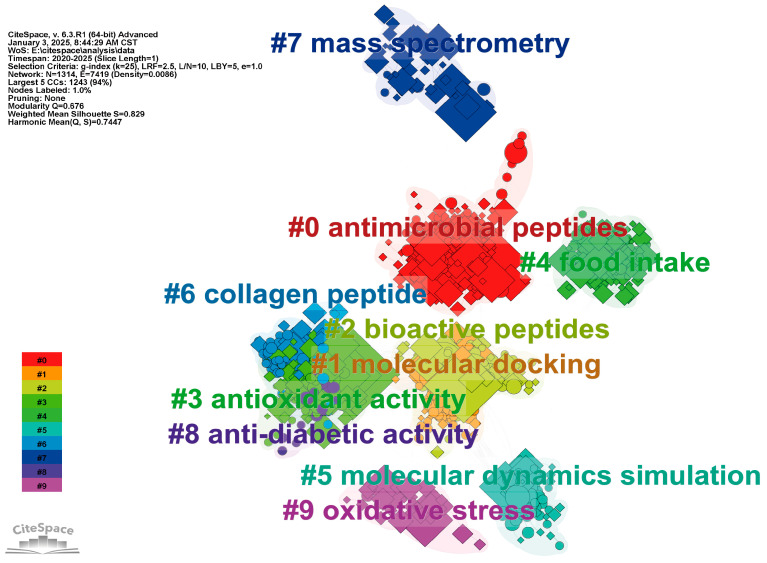
Research progress of food source peptides in the past 5 years. Different colors indicate different clusters, and the top 10 most researched directions or fields are listed according to keywords and references. Figure made by CiteSpace (6.3. R1).

As of 2 January 2025, a search using the terms “food” and “peptides” for research papers from 2020 to 2025 in the Web of Science Core Collection was conducted. A total of 17,260 relevant documents were retrieved, and Citespace (6.3. R1) was used for cluster analysis [8]. The final analysis cluster diagram (Figure 1) was obtained, with a modularity (Q) of 0.676 and a weighted mean silhouette (S) of 0.829. Generally, a Q value greater than 0.3 indicates a significant clustering structure, and an S value greater than 0.7 suggests convincing clustering results. The clustering results showed that recent studies on peptides in food are categorized into activity exploration (#0, #3, #8, #9), peptide activity prediction (#1, #5), peptide identification (#7), and peptide sources (#4, #6). Antimicrobial resistance represents a paramount global health challenge. Antimicrobial peptides (AMPs), recognized for their polypharmacological profiles (exhibiting antibacterial, antifungal, and antiviral efficacy) and low resistance induction potential, stand as leading candidates to supersede conventional antibiotics [9]. Nevertheless, a critical knowledge gap persists regarding the “activity cliff” phenomenon, wherein amino acid sequence variations provoke drastic reductions in antimicrobial potency. To address this, integrating computational biology with machine/deep learning frameworks is imperative to transcend linear structure–activity relationship paradigms and elucidate non-linear sequence-function mappings. Concurrently, cell-penetrating peptides (CPPs) are extensively engineered as modular platforms for intracellular translocation of membrane-impermeable cargoes, establishing them as versatile scaffolds for next-generation biotherapeutics and drug delivery systems [10]. Notably, food-derived casein hydrolysates (<1600 Da) have demonstrated epithelial transport competence, facilitating their entry into systemic circulation—a property exploitable for bioactive peptide design [11]. Since this review only lists the top 10 research clusters, other topics such as gut–peptide interactions [12], peptide homeostasis [13], and peptide delivery and release [14] are not discussed despite the numerous research reports. The use of molecular simulation techniques to obtain potentially bioactive peptides has been integrated into these hot-spot clusters. This indicates that besides the traditional cell and animal experiments, computer simulation calculations based on principles such as Newtonian mechanics and quantum mechanics have become a reliable technical means for researchers to screen bioactive peptides in high throughput [15,16]. Additionally, the source materials, production strategies, and application scope of evidence-supported bioactive peptides require focused attention [17].

## 3. Main Source and Function of Bioactive Peptides

**Figure 2:**
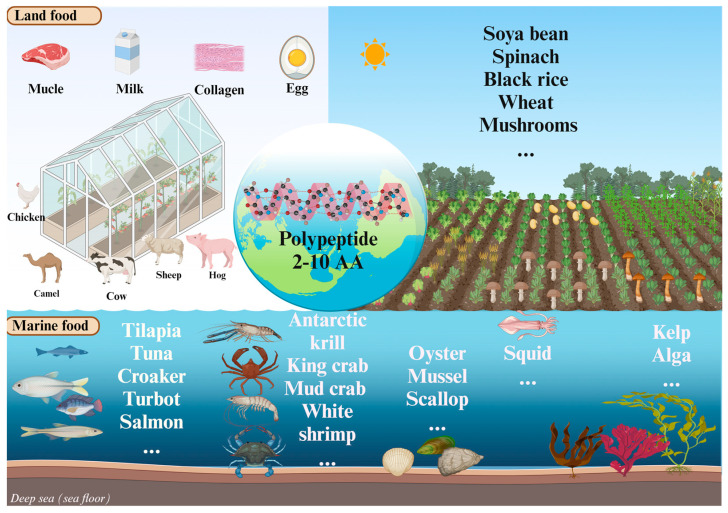
Partial distribution of species producing different functional bioactive peptides. Aquatic products do not distinguish between seawater and fresh water. Figure made by BioRender (agreement number: WL28ICF78U).

There is a vast array of proteins in food, which not only supplement the amino acids and energy required by the human body but also constitute a huge peptide code library. Currently, the “BIOPEP-UWM” database has collected more than 5000 bioactive peptides. The main sources of proteins in food include animal and plant sources. Animal sources include milk, fish, shrimp, shellfish, pork, beef, porcine skin, and cowhide. Plant sources are soybeans, spinach, mushrooms, wheat, and black rice, among others (Figure 2).

### 3.1. Animal Protein

#### 3.1.1. Aquatic Food

Aquatic foods encompass more than 80% of the world’s biological resources, such as fish, shrimp, shellfish, and cephalopods [18]. It is projected that by 2030, the protein contribution from aquatic foods may account for more than 13% of the total protein consumed [19]. Consequently, this vast protein source provides substantial support for exploring and preparing related bioactive peptides. Other researchers have already provided a comprehensive summary of the bioactive peptides produced by different species of aquatic products and their by-products [20]. In fish, whether freshwater or marine, a large number of bioactive peptides have been reported, such as tilapia (*Oreochromis niloticus*) [21] and hairtail (*Trichiurus japonicus*) [22].

The global production of L. vannamei has been on the rise, with the global output reaching 5.8 million tons in 2020, more than 50 times the 155,000 tons produced in 2000 [23]. Shrimp by-products, particularly shrimp heads, are rich in enzymes and proteins, offering advantages for obtaining a variety of peptides [24]. Studies have reported that protein hydrolysates from shrimp by-products possess anti-bacterial, ACE activity inhibitory, and anti-cancer properties [25,26]. Additionally, a small peptide (5 kDa) extracted from the acetone powder of the red king crab hepatopancreas has been shown to exhibit antibacterial activity against *M. lysodeikticus* [27]. Mao et al. [28] utilized molecular docking to identify new xanthine oxidase (XO) inhibitory peptides (AEAQMWR) in swimming crabs or Pacific white shrimp.

The catch of aquatic products such as coral, pearls, shellfish, and seaweed was approximately 7700 tons in 2024 [29]. Typical shellfish include oysters and mussels. The annual production of oysters is about 6.1 million tons [30]. Oysters have a rich protein content (39.1% to 53.1%) [31]. Oyster peptides have been reported to have antioxidant [32] and immune-modulating [33] properties. Oyster peptides have become one of the main deep-processing products of oysters [34].

#### 3.1.2. Terrestrial Food

The global meat production in 2024 was expected to reach 373 million tons (calculated by carcass weight) [29]. These farmed animals, including bones, offal, and hides, have been utilized comprehensively. However, these by-products are still largely most utilized in low-value processing, such as in pet food and chemical products. It is necessary to explore potential substances of higher value further. Exploring bioactive peptides is well-suited to higher-value processing and utilization of these by-products.

Large animals such as pigs, cattle, and sheep provide a substantial amount of muscle tissue for human consumption and offer a rich source of protein from their abundant internal organs and skin. Studies have explored the antioxidant and xanthine inhibitory activities of peptides derived from the hydrolysis of proteins in the heart, liver, and lungs [35,36]. Animal skin contains a large amount of collagen, and the hydrolyzed collagen peptides exhibit antioxidant, anti-inflammatory, and skin-health-promoting activities [37]. Otherwise, marine animals also contain collagen, such as the hydrolyzed peptides from fish swim bladders, which also possess bioactivity [38]. In novel food processing, these peptides are also commonly used in applications such as 3D food printing [39], umami peptide discovery [40], and protein-stabilized emulsion gels [41].

Milk is an essential component of the diet and a vital nutritional source for newborn mammals. It is rich in vitamins, minerals, hormones, enzymes, growth factors, oligosaccharides, bioactive peptides, and nutrients [42]. Bioactive peptides in milk are categorized into endogenous and exogenous hydrolytic peptides [42]. In recent years, endogenous peptides have been found to possess immune-modulating, hypoglycemic, and antioxidant functions [43,44], playing a crucial role in protecting newborns from diseases, especially in breast milk [45]. Exogenous hydrolytic peptides are beneficial hydrolytic peptides from animal milk proteins, such as beta-casein, α-S1-casein, α-S2-casein, kappa-casein, and whey protein [46]. These animal milks primarily come from cows, goats, sheep, camels, and donkeys [42]. Notably, milk is commonly regarded as a food that aids sleep. Drinking a glass of milk before bed helps with falling asleep. This is attributed to its psychological suggestion (the memory of being fed by a mother before sleep) and the rich sleep-promoting compounds it contains, such as tryptophan. Research indicates that milk collected at night contains a high amount of tryptophan and melatonin, which extend sleep duration and have anxiolytic effects [47].

Eggs are considered a high-quality protein source, with egg whites accounting for about 63% of the total weight [48]. More than a hundred proteins have been identified in egg whites, with ovalbumin, ovotransferrin, lysozyme, and ovomucin well-characterized [49]. These proteins themselves possess bioactive properties. For instance, lysozyme has antibacterial activity and is commonly used as an oral antibacterial agent and preservative [50]. Ovotransferrin has been studied for its potential use in treating inflammatory bowel disease [51]. Moreover, peptides derived from these proteins in eggs also exhibit antioxidant and anti-inflammatory activities, potentially reducing oxidative stress and inflammation-related chronic diseases [52]. For AMPs, a comprehensive database has been compiled, with milk-derived peptides that not only exhibit antimicrobial but also antifungal activities [53].

### 3.2. Plant Protein

Plant proteins are relatively more abundant and cheaper than animal proteins, but their use is still limited, mostly as animal feed to produce meat, eggs, and milk. The conversion rate of plant proteins to animal proteins is only about 3% [54]. Therefore, plant proteins are also an excellent choice for exploring bioactive peptides. For example, after simulated in vitro gastrointestinal digestion, soybeans yield peptides with antioxidant activity [55]. Otherwise, chickpeas (ANDISFNFVRFNETNLILGG, RQSHFANAQP), lentils (EVASYSGW, FFADTGIK), and other legume protein peptides have been found to have known anti-breast cancer functions [56]. Through virtual enzymatic hydrolysis with eight enzymes and validation experiments, rapeseed protein has identified three novel ACE inhibitory peptides: FQW, FRW, and CPF [57]. Seaweed also has a variety of biological potentials, and its protein-derived peptides have anti-hypertensive and anti-cancer effects [1,58].

## 4. Receptor Protein

**Figure 3:**
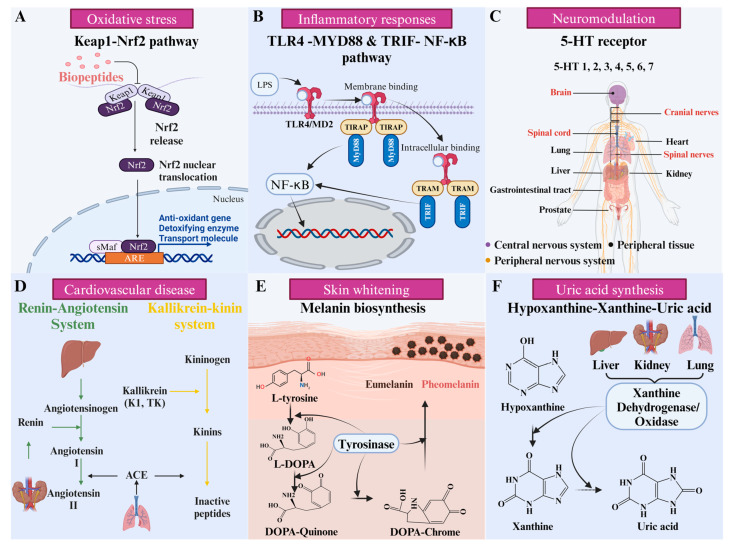
The mechanism or distribution of action of protein receptors. (**A**) Keap1-NrF2 oxidative stress pathway involved in keap1; (**B**) MYD888 and NF-κB in which TLR4 is involved; (**C**) distribution of 5-HT receptors in human organs; (**D**) renin–angiotensin system and kallikrein–kinin system in which ACE participates; (**E**) melanin biosynthesis involving tyrosinase [59]; (**F**) part of purine dehydrogenase metabolic pathways involved in xanthine dehydrogenase/oxidase. Figure made by BioRender (agreement number: SZ28ICEP4K).

The functional activities of peptides are often closely related to specific protein receptors in the human body. Identifying protein receptors using molecular modeling techniques is important in identifying this (sub)class of bioactive peptides. This section reviews the interaction mechanisms of targeted protein receptors and bioactive peptides in antioxidant, antidepressant, anti-inflammatory, blood pressure regulation, inhibition of melanin production, and uric acid synthesis (Figure 3).

In addition, the protein receptor PDB ID that is mainly used is summarized in Table 1. In studying receptors, attention should be paid to the receptor protein source, the resolution quality, and the small molecular ligand contained in the structure. Random selection of receptor proteins leads to implausible conclusions.

### 4.1. Keap 1

Keap1 is a major negative regulator of cellular protective gene expression and targets the inhibition of Nrf2-dependent gene expression [60] (Figure 3A). Studies on cardiovascular events such as atherosclerosis and myocardial infarction induced by oxidative stress suggest that Keap1, a negative regulator of Nrf2, may be one of the target proteins [61,62]. The Keap1-Nrf2 pathway is a key pathway for clearing oxidative stress in the body. When Keap1 is subjected to external oxidative stimulation, it detaches from the Nrf2 factor. After nuclear translocation, the Nrf2 factor binds to the antioxidant response element (ARE), leading to the expression of heme oxygenase-1, thereby activating the antioxidant stress system and regulating the expression of various antioxidant enzymes in the cell, such as catalase, superoxide dismutase, and glutathione peroxidase. Peptides bind to Keap1 with high affinity, preventing its interaction with Nrf2, promoting the further transmission of the Nrf2 factor, and enhancing the cell’s antioxidant capacity. Recent studies have found that oligopeptides (ARNF, QADF, ARRF) derived from the enzymatic hydrolysis of bovine hemoglobin with trypsin (EC 3.4.21.4), pepsin (EC 3.4.23.1, pH 1.3), and papain (EC 3.4.22.2) show good interaction with Keap1. Further cell experiments have proven that ARRF and ARNF improve the oxidative stress and inflammation of RAW264.7 cells [63].

### 4.2. Toll-like Receptor 4 (TLR4)

TLR4 is a pattern recognition receptor essential to the mammalian innate immune system (Figure 3B). It recognizes lipopolysaccharides from Gram-negative bacteria or bacterial endotoxins. The dimeric complex formed upon recognition simultaneously activates the MYD88 and TRIF-dependent pathways, ultimately activating the downstream NF-κB signaling pathway, which induces inflammation [64]. Overactivation of TLR4 triggers the production of various inflammatory factors and is associated with conditions such as sepsis, endotoxemia, acute lung injury, rheumatoid arthritis, and cardiovascular diseases. Therefore, inhibiting TLR4 has the potential to exert anti-inflammatory effects. Research has shown that oligopeptides derived from the enzymatic hydrolysis of bovine hemoglobin (ARNF, RRF, ARRF) have strong interactions with TLR4 and exhibit anti-inflammatory effects [63]. Another study found that peptides derived from hemp (*Cannabis sativa* L.) protein (DDNPRRF, SRRFHLA, RNIFKGF, VREPVFSF, QADIFNPR, and SAERGFLY) also have a high affinity for TLR4 and serve as a source of peptides for preventing inflammation-related diseases [65].

### 4.3. 5-Hydroxytryptamine (5-HT) Receptor

Serotonin (5-hydroxytryptamine, 5-HT) is derived from the hydroxylation and decarboxylation of tryptophan (Figure 3C). It is a bioamine that plays a crucial role in cardiovascular function, gastrointestinal motility, and mental disorders [66]. Serotonin exerts its physiological functions through seven types of serotonin receptors, including 5-HT1, 5-HT2, 5-HT3, 5-HT4, 5-HT5, 5-HT6, and 5-HT7 [67]. These receptors are primarily distributed in the central nervous system and peripheral tissues. The 5-HT1A and 5-HT2A receptors play particularly significant roles in neuroregulation. The 5-HT1A receptor mainly acts as an auto receptor for 5-HT neurons, inhibiting the release of 5-HT through a negative feedback mechanism, thereby regulating 5-HT neurotransmission. This regulatory function is essential for maintaining the homeostasis of the nervous system. Activation of the 5-HT2A receptor increases neuronal excitability, especially in the cortex and hippocampus. This increased excitability is associated with improved cognitive function and mood regulation. Research has found that YLG produces anxiolytic-like effects by activating 5-HT1A, dopamine D1, and GABAA receptors [68]. Moreover, the dipeptide YL has been found to have antidepressant activity when administered orally, intracerebroventricularly, and intraperitoneally, and it also increases the number of c-Fos-expressing cells in the dentate gyrus of the hippocampus [69]. Since many 5-HT receptors are located in the brain, food-derived peptides must pass through the intestinal and blood–brain barriers to reach these receptors, making it difficult for bioactive peptides to act on brain receptors directly. Nevertheless, bioactive peptides also promote the synthesis of 5-HT by enterochromaffin (EC) cells in the gut, increasing the levels of 5-HT in peripheral tissues and thereby regulating peripheral physiological functions [69].

### 4.4. Angiotensin I-Converting Enzyme (ACE)

ACE (EC 3.4.15.1) has been extensively studied as a therapeutic target for cardiovascular and cerebrovascular diseases, particularly hypertension. ACE regulates blood pressure through the renin–angiotensin and kallikrein–kinin systems [70] (Figure 3D). ACE catalyzes the conversion of the inactive hormone precursor angiotensin I to the active hypertensive hormone angiotensin II and plays a role in the degradation of the vasodilator bradykinin. The inhibition of ACE by bioactive peptides is either competitive or non-competitive. Peptides derived from peanut protein have been reported to bind competitively to the substrate, while purified peptides from mushrooms exhibit non-competitive inhibition [71]. Numerous reports of ACE-inhibitory peptides are derived from animal and plant proteins. For example, peptides from rapeseed protein (FQW, FRW, CPF) [57] and milk (DGG, DGGM) [72] have been identified as having ACE-inhibitory activity.

### 4.5. Tyrosinase

Melanin absorbs ultraviolet rays and is a natural endogenous functional substance, including eumelanin, pheomelanin, and allomelanin (Figure 3E). However, excessive deposition of melanin leads to various skin problems, such as melasma, age spots, and melanoma. Reducing melanin can brighten and whiten the skin. Tyrosinase (EC 1.14.18.1) is a copper-containing oxidoreductase produced by melanocytes and is a key enzyme in melanin synthesis [73]. It catalyzes the oxidation of tyrosine to dopa (DOPA), which is further oxidized to dopaquinone, ultimately forming melanin. Tyrosinase has become a major target for inhibiting melanin formation. Currently, food-derived proteins such as fish, papaya seeds, sesame, and egg white protein have been found to contain peptides with good tyrosinase inhibitory activity, which are used to develop functional foods beneficial to skin health [73].

### 4.6. Xanthine Oxidoreductase (XOR)

XOR comprises two forms: xanthine dehydrogenase (XDH) and xanthine oxidase (XO) (Figure 3F). XOR in mammals is a homodimer, with each subunit containing three domains. XOR catalyzes the oxidation of hypoxanthine to xanthine and further to uric acid. XDH primarily executes the purine catabolism step from hypoxanthine to uric acid, mainly using NAD+ as the electron acceptor. The conversion of xanthine dehydrogenase to XO occurs through the oxidative modification of thiols. Therefore, XO is referred to as the isomer of XDH. In addition to its role in purine catabolism, XO also catalyzes the transfer of monovalent and divalent electrons to O_2_, generating superoxide ions and hydrogen peroxide [74]. In recent years, xanthine oxidase has been a key target for treating hyperuricemia due to its role as the rate-limiting enzyme in uric acid production and as a reactive oxygen species-generating enzyme. Excess uric acid levels cause inflammation, leading to gouty arthritis. Clinically, reducing uric acid levels involves inhibiting the synthesis of uric acid and decreasing the intake of high-purine foods. Food-derived protein peptides also contain a rich array of XO inhibitory peptides, whether prepared by enzymatic digestion or produced by human gastrointestinal digestion. For example, three XO inhibitory peptides (ALSGSW, GGYGIF, MAIGLW) have been identified in oysters [75], and novel XO inhibitory peptides (DGG, DGGM) from lactoprotein hydrolyzed by gastrointestinal peptidases have been screened [72].

## 5. Computer-Based Screening of Active Peptides

Computational approaches for bioactive peptide screening primarily involve three key scenarios: virtual enzyme digestion, bioactivity screening, and bioactivity prediction. Table 2 summarizes the comparative advantages and disadvantages of these different computational methods. 

Virtual enzyme digestion: This method comprehensively accounts for all potential amino acid sequences generated during enzymatic cleavage. However, the impact of digestion parameter settings on the final peptide profile composition requires further investigation. 

Bioactivity screening via molecular docking: Serving as a powerful tool for the rapid preliminary screening of bioactive peptides, docking approaches necessitate careful selection of both the docking methodology and precision level based on the specific requirements of the target task. 

Mechanistic studies via molecular dynamics (MD) simulation: Used to investigate the binding mechanisms and dynamics of bioactive peptides over timescales, MD simulations require a critical balance between computational accuracy and resource efficiency. 

Bioactivity prediction models: Advanced prediction models significantly accelerate the mining (discovery/identification) of bioactive peptides. However, constructing a model with high predictive accuracy and robust generalization capabilities requires a foundational dataset of substantial size and high quality. Predictive modeling is primarily categorized into classification and regression problems. For peptide activity prediction: Classification models output the probability of a peptide possessing specific bioactivity, determining its functional potential; regression models predict continuous numerical values (e.g., IC_50_, binding affinity coefficients), with prediction deviations quantified by root mean square error (RMSE) [76]. State-of-the-art deep neural networks (e.g., transformer, convolutional neural network) achieve >0.9 area under the ROC curve (AUC) [77].

### 5.1. Virtual Enzymolysis

**Figure 4:**
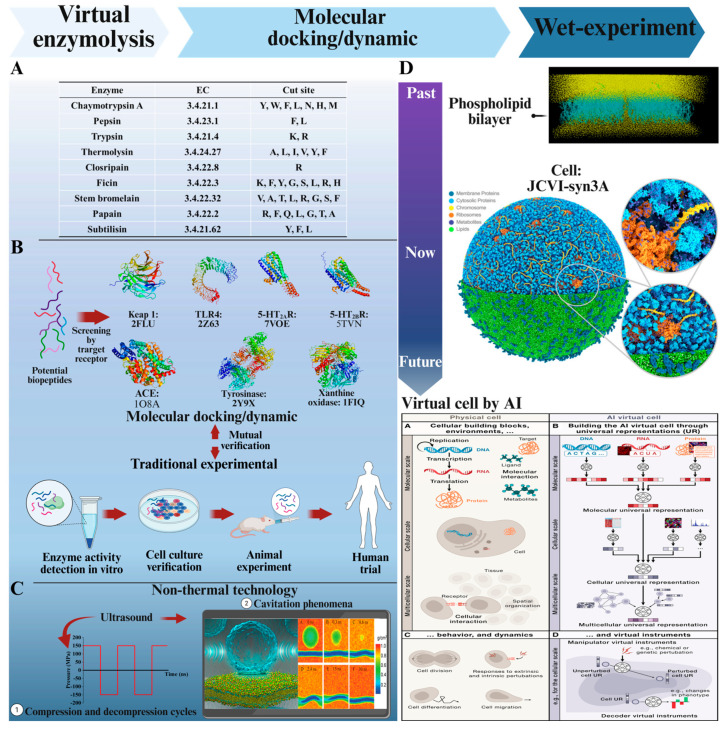
Overview of virtual enzymolysis, molecular docking, molecular dynamics simulation, construction of non-thermal processing techniques, and virtual cell models. (**A**) Common enzymes used in virtual enzymatic hydrolysis and their cleavage sites on amino acid sequences. (**B**) The mutual verification of traditional experimental and molecular simulation for bioactive peptide mining. (**C**) The construction of non-thermal processing techniques in molecular simulation, taking ultrasonic processing as an example (reproduced with permission from Fu et al., *Journal of Physical Chemistry Letters*; published by American Chemical Society 2015) [78]. (**D**) The development process of simulated organelles: from the past (lipid bilayer) to the future (AI organs) (some pictures obtained from [3,79,80]. Reproduced with permission from Shamloo et al., *Journal of Magnetism and Magnetic Materials*; published by Elsevier 2016 [79]. Figure made by BioRender (agreement number: IN28NA81P9).

Virtual enzymatic digestion is the first step in virtual screening (Figure 4A). It is a method that uses computer simulation techniques to predict and analyze the hydrolysis process of proteins or peptides under the action of specific enzymes. This technology predicts the possible peptide segments produced by simulating the interactions between enzymes and substrates, thereby providing a theoretical basis for experimental design and the screening of bioactive peptides. Virtual enzymatic digestion quickly predicts the bioactive peptides that may be produced after protein hydrolysis, thus narrowing down the experimental screening range. The working principle is based on the identified enzyme cleavage sites used to recognize target amino acid sequences and hydrolyze specific peptide bonds [2].

The protein amino acid sequence and the enzyme specificity are critical parameters in virtual enzymatic digestion. The protein amino acid sequence is essential for obtaining bioactive peptide sequences. If the amino acid sequence of the protein of interest has not been studied, it will be necessary to use methods such as gene sequencing and translation or protein mass spectrometry analysis to obtain the amino acid sequence of the target protein [81].

Enzymes such as chymotrypsin A (EC 3.4.21.1), pepsin (EC 3.4.23.1), trypsin (EC 3.4.21.4), thermolysin (EC 3.4.24.27), clostripain (EC 3.4.22.8), ficin (EC 3.4.22.3), stem bromelain (EC 3.4.22.32), papain (EC 3.4.22.2), and subtilisin (EC 3.4.21.62) are commonly used in industry and have well-defined cleavage sites. The diversity of cleavage sites is crucial in determining the function and activity of protein-derived peptides. Therefore, discovering more enzymes with unique cleavage sites and safety profiles is significant for exploring bioactive peptides. Currently, three main high-throughput enzyme screening strategies exist: display-based, cell-based, and computer-simulation-based [20]. Computer simulation-based screening enables enzyme engineering (thermal stability and solvent tolerance enhancement), function prediction (substrate specificity and catalytic mechanism identification), enzyme design (designing enzymes with specific functions and site directed mutagenesis), and enzyme substrate binding mode exploration [82].

### 5.2. Molecular Docking/Dynamics Simulation Combined with Wet-Lab Experiments

Molecular simulation encompasses molecular docking and molecular dynamics simulation, two complementary techniques primarily based on computer platforms capable of recognizing molecular structures. Molecular docking uses static 3D structures to quickly predict binding patterns between small and large molecules, optimizing energy states to minimize energy, following the principle of minimal energy. In 1957, Alder and Wainwright pioneered molecular dynamics studies with a 32-particle hard sphere system [83]. Since then, molecular dynamics simulation has been widely applied, elucidating the dynamic changes in the conformation of molecules under various conditions over a certain timescale [4]. The application of molecular simulation methods helps to save a significant amount of experimental materials, reduces the time and effort of repetitive experiments, and allows for direct analysis of the changes and interactions of macromolecules from an atomic perspective [84]. In the traditional approaches to mining bioactive peptides, the extracted and purified peptides must undergo target enzyme activity testing (inhibition/activation), cell culture tests (toxicology, gene expression, etc.), animal model validation, and human trials. Incorporating computer-based molecular simulation has added new pathways to this traditional experimental process (Figure 4B).

An increasing number of scholars are combining molecular simulation with traditional wet-lab validation to analyze and verify the mechanisms of action of bioactive peptides from multiple perspectives. Based on the analysis of recent studies (Table 3), researchers have divided the application of molecular simulation-related technologies into two directions: (1) using molecular simulation as one of the means for screening bioactive peptides; and (2) using it as a tool for analyzing the molecular mechanisms of peptide-protein receptor interactions. In Direction 1, the main approach is to utilize known protein peptide sequences or to identify peptide sequences from scratch using LC-MS/MS, and then to use molecular docking and other online prediction tools (with predict functions such as solubility, stability, and bioactivity) for high-throughput screening of bioactive peptides. Focusing on physicochemical parameters and combined experimental and computational approaches can help to identify novel antimicrobial and anticancer peptides [85]. Subsequently, target peptides are synthesized, and related enzyme, cell, and model mouse validation experiments are conducted to further prove their functional activity and explore their dose–effect relationships [65,72]. Direction 2 involves using molecular simulation to investigate the molecular mechanisms of peptide–protein receptor interactions in traditional peptide mining, which is crucial for elucidating the structure–activity relationship of peptides [46].

Compared to wet-lab experiments, virtual screening offers superior screening efficiency, larger-scale screening capacity, and lower screening costs. Based on current supercomputing platform pricing, screening 10,000 peptides for a potential activity by autodock vina software incurs approximately 8000 core hours of computational expense (roughly USD 60). In contrast, wet-lab experimentally validating the activity of 10,000 peptides (5–10 amino acid residues) requires 10,000 repetitions of high-purity peptide synthesis and verification assays. Consequently, the stark contrast in time and material costs is undeniable. It should be noted, however, that virtual screening results must undergo necessary wet-lab validation. For example, while the DeepAMP model achieved an R2 of 0.82 for predicting antimicrobial peptides against E. coli, its predictions still require experimental verification [86].

This integrated technology is primarily employed for preliminary screening and validation of peptide bioactivity. However, its industrial-scale implementation necessitates prioritizing production cost structures and processing adaptability, as obtaining high-purity target peptides—typically achieved through solid-phase synthesis in academic research—requires synergistic application of precision enzymatic hydrolysis and directed enrichment technologies for food manufacturing. Notably, AI-powered discovery platforms like Nuritas’ Magnifier NπΦ™ have successfully translated bioactive peptide research into commercial ingredients, as evidenced by market-ready products including PeptiStrong™, PeptiYouth™, and PeptiSleep™ (https://www.nuritas.com).

**Table 3:** Research report of molecular simulation combined with wet-lab experiment for mining foodborne active polypeptides.

Protein Sources	Peptides Sequence	Bioactive	Molecular Simulation	Wet-Lab Experiment	References
Porcine visceral proteins	3–6 amino acid residue	Prevent xanthine oxidase (XO)-mediated hyperuricemia	Docking receptor: XO (PDB ID: 3NVY)	XO inhibitory activity in vitro	[36]
Rapeseed proteins	FQW, FRW, and CPF	angiotensin I-converting enzyme (ACE) inhibitory	Docking receptor: ACE (PDB: 1O8A)	ACE inhibitory activity in vitro	[57]
Porcine bone collagen	GPGPM, RGPPGPM, PSGGF, VGGF, FSGL, PGSPGPGPR, AGPGPM and GPTGF	Anti-inflammatory	Docking receptor: calcium-sensing receptor (PDB: 7DD7)	(1) Scavenge 2,2′-azinobis-(3-ethylbenzthiazoline-6-sulphonate) (ABTS) radical ability (2) Fe^2+^ chelating activity (3) Albumin inhibitory activity (4) NO inhibitory activity (5) Cytotoxicity test (6) Determination of inflammatory cytokines	[87]
Porcine offals (heart, liver and lung) and one muscle the Longissimus Dorsi	\	ACE inhibitory	Virtual enzymatic hydrolysis by BIOPEP (proteinase K, papain, subtilisin, bromelain, and ficin)	(1) ACE inhibitory activity in vitro (2) Determination of oxygen radical absorbance capacity (3) Determination of the inhibitory activity of dipeptidyl peptidase 4	[35]
Swim bladders of monkfish (*Lophius litulon*)	SEGPK, FDGPY and SPGPW	ACE inhibitory	Docking receptor: ACE	(1) ACE inhibitory activity in vitro (2) Effects on human umbilical vein endothelial cells	[38]
Bovine milk proteins: α-lactalbumin (P00711), β-lactoglobulin (P02754), β-casein (P02666), α-S_1_-casein (P02662), α-S_2_-casein (P02663), κ-casein (P02668), lactoferrin (P24627)	DGG, DGGM	ACE and XO inhibitory	(1) Virtual enzymatic hydrolysis by ExPASy Peptide Cutter (pepsin, trypsin, and chymotrypsin) (2) Docking receptors: ACE (PDB ID: 1O8A) and XO (PDB ID: 1FIQ) (3) Molecular dynamics simulation	(1) Determination of antioxidant activities (2) 2,2-Diphenyl-1-picrylhydrazyl (DPPH) radical scavenging activity (3) ABTS radical scavenging activity (4) Hydroxyl radical scavenging activity (5) ACE inhibitory activity in vitro (6) XO inhibitory activity in vitro	[72]
Pacific white shrimp and swimming crab	17 novel XO inhibitory peptides (AEAQMWR exhibited the greatest XO inhibitory activity in vitro)	Anti-hyperuricemic activity	Docking receptor: XO (PDB ID: 1N5X)	(1) Assessment of XO inhibitory activity of sample in vitro (2) HK-2 cells experiment (3) Analysis of inflammation levels (IL-6, IL-1β, and TNF-α)	[28]
Hemp (*Cannabis sativa* L.) protein	DDNPRRF, SRRFHLA, RNIFKGF, VREPVFSF, QADIFNPR and SAERGFLY	Immunomodulatory activity	(1) Docking receptor: TLR4/MD2 complex (PDB: 3VQ2) (2) In silico analysis (Peptide Ranker, AnOxPePred 1.0, PreAIP, PlifePred, HLP)	(1) CACO-2 cells viability assay (2) Analysis of inflammation levels (TNFα, IL-1β, IL-4, IL-6, IL-10, and TLR4)	[65]
Bovine hemoglobin	ARRF and ARNF	Antioxidant and anti-inflammatory	(1) Virtual enzymatic hydrolysis by ExPASy Peptide Cutter (trypsin, pepsin, papain) (2) Prediction of peptides (PeptideRanker, ToxinPred, INNOVAGEN, A11erTOP) (3) Docking receptors: TLR4 (PDB ID: 2Z63) and Keap1 (PDB ID: 2FLU) (4) Molecular dynamic simulation of peptides and receptors	(1) RAW264.7 cells experiment (2) Determination of the contents of NO, TNF-α, IL-6, and PGE2 (3) Etermination of ROS content, SOD, MDA, and GSH-Px levels	[63]
Hairtail (*Trichiurus japonicus*)	VVFEVFW	Antidepressant	(1) Docking receptor: monoamine oxidase A (MAO-A) (PBD ID: 2Z5X)	(1) MAO-A inhibition assay	[22]
Egg white proteins including ovalbumin, ovotransferrin, and ovomucoid	GDVA and DEK	Tyrosinase inhibitory	(1) Virtual enzymatic hydrolysis by ExPASy Peptide Cutter (chymotrypsin, trypsin, pepsin) (2) Prediction of peptides (peptide property calculator, ToxinPred) (3) Docking receptor: tyrosinase (PDB ID: 2Y9X) (4) Molecular dynamic simulation of peptides and receptors	(1) Tyrosinase inhibitory activity in vitro	[73]
Oyster protein	ALSGSW, GGYGIF, and MAIGLW	XO inhibitory	(1) Docking receptor: XOD complex (PDB ID: 1N5X)	(1) Determination of inhibitory activity of XO	[75]
Kuruma shrimp (*Marsupenaeus japonicus*) heads	ARL/I (ACE inhibitory)	ACE inhibitory	(1) Docking receptor: ACE (PDB ID: 1O86)	(1) ACE inhibitory activity (2) Determination of ACE inhibition pattern (3) Hemolytic activity assay	[26]

### 5.3. Virtual Organelles/Cells Used for Screening Active Peptides

Peptides, especially some oligopeptides with 2–8 amino acid residues, can cross the blood–brain barrier, and act on receptors in brain tissue [88]. The blood–brain barrier (BBB) is a specialized physiological barrier between the brain’s blood vessels and brain tissue, composed of capillary endothelial cells, the basement membrane, pericytes, and astrocytes. It protects the central nervous system from harmful substances in the blood and maintains the stability of the brain’s internal environment [89]. However, this BBB function also poses a significant barrier to drugs entering brain tissue. Bioactive peptides that target receptors such as 5-HT2AR in the brain must pass through the BBB. In vitro models for simulating the BBB include microfluidic chips and stem cell-derived endothelial cell models [90]. Two gold-standard measures for evaluating BBB permeability are logBB (the concentration of the drug in the brain divided by the concentration in the blood) and logPS (permeability surface-area product). However, both methods are time-consuming and expensive.

Computational simulation methods offer viable alternatives (Figure 4D). Since the pioneering work of Young et al. [91] using quantitative structure–activity relationship (QSAR), computer predictions of drug permeability through the BBB have advanced. By constructing a 1,2-dioleoyl-sn-glycero-3-phosphocholine bilayer model to simulate the BBB, it has been shown that computational predictions correlate well with experimental values, with R2 greater than 0.90 [92]. Another study described the interaction between magnetic nanoparticles and the cell membrane by constructing a phospholipid bilayer, bridging the gap between nano and microscale models of the BBB [79]. However, computational simulation methods have some obvious limitations, including the inability to account for any non-passive transport, using a single type of lipid without considering mixed lipid systems, and the cell gap.

Biological molecular functions arise from molecular interactions within cellular environments. To comprehend the changes in interactions at the molecular or even atomic level, molecular dynamics (MD) simulations serve as a viable alternative. With the advancement of central processing units (CPUs) and graphics processing units (GPUs), MD simulations have expanded to encompass systems with hundreds of millions of atoms, such as human nuclear pores [93] and atomic models of cytoplasm [94]. Further progress has enabled the successful simulation of an entire cell, JCVI-syn3A [80]. Traditionally, the biological research community has relied on computational models to analyze past experimental data based on existing hypotheses. In the future, virtual cells could transform paradigms using computer simulations to explore possible hypotheses. Bunne et al. [3] proposed creating artificial intelligence (AI) virtual cells based on omics, constructing a large-scale neural network model that simulates the behavior of molecules, cells, and tissues under various conditions. Computational simulation replicates cellular behavior, providing predictive capabilities for drug development and biological research. These ‘virtual cells’ are designed to learn the relationship between cellular state and function, predicting the consequences of perturbations—such as gene knockdown or drug application—across diverse cell types and cellular contexts. Currently, this field is gaining significant traction due to advancements in large-scale single-cell measurement technologies, the availability of richer perturbation datasets, and progress in machine learning. For example, the recently launched Virtual Cell Challenge (hosted at https://virtualcellchallenge.org) further drives progress towards the goal of predicting cellular responses to perturbations [95]. These breakthroughs in computing undoubtedly offer a faster and more cost-effective screening method for the high-throughput exploration of bioactive peptides in food. In the future, AI models may be able to directly predict all potential targets, diseases, and cellular interactions of bioactive peptides resulting from human gastrointestinal digestion.

## 6. Computer-Based Experimental Design for Bioactive Peptide Preparation

### 6.1. Application of Non-Thermal Processing Techniques in the Preparation of Active Polypeptides

The previous review described various non-thermal processing methods to explore bioactive peptides, such as ultrasound, high-pressure processing, electric field, and magnetic field [96]. In peptide preparation experiments, high-pressure processing and ultrasound have been used to extract bioactive peptides and improve their functional properties [97]. Ultrasound-assisted enzymatic hydrolysis of porcine bone collagen to prepare anti-inflammatory peptides has been shown to increase the content of peptides and α-helices in the peptides, enhancing their free radical scavenging and iron ion-chelating abilities [87]. Additionally, low-intensity ultrasound has been found to enhance the ability of peptides to sterilize Escherichia coli, with the principle being that ultrasound has a slight untwisting effect on the TGH2 peptide, thereby promoting the interaction between the peptide and the E. coli cell membrane [98]. High-pressure processing has also been used to increase the efficiency of enzymatic hydrolysis of food proteins and enhance hydrolyzed peptides’ bioactivity and yield [97]. These techniques ultimately involve changes in the physical field, including changes in pressure, voltage, magnetic force, sound waves, etc. These changes are simulated in molecular simulations through alterations in the direction of the force and periodic variations to achieve a realistic simulation of the action characteristics (Figure 4C).

In fact, there are few reports on the use of molecular simulation to construct non-thermal processing technologies to study their impact in the food field, even though computer hardware and software have been significantly upgraded. Researchers have found that under pressure conditions, the AK16 peptide and a C-peptide analog form more helical structures and the salt bridge of the C-peptide analog is broken under high pressure [99]. Another study reported the effects of ultrasonic field on the conformation and aggregation patterns of walnut protein in all-atomic molecular dynamics and coarse-grained molecular dynamics, and explored the mechanism of improving the functional properties of walnut protein from the perspective of molecular simulation [100].

### 6.2. Non-Thermal Processing Techniques Constructive in MD Simulation

Force fields are widely used in molecular dynamics simulation software like GROMACS and AMBER. Their specific principles are well explained in the software manuals. Pressures are divided into static pressure technology and dynamic pressure technology. In molecular dynamics simulations, the building of pressure involves specifying both the direction of its application and controlling its magnitude.

According to the characteristics of ultrasonic action, there are mainly two ways to construct it in molecular simulations. One application of ultrasound is the compression and decompression cycles [101], achieved by applying sinusoidal or square wave variations to the pressure system. Another is the stable or inertial cavitation phenomena induced by ultrasound [102]. Common simulation amplifications include locally increasing temperature [103] by removing a small number of water molecules from the system to form a complete spherical space and applying negative pressure cycles to the molecular system under study. Stable cavitation is modeled through low-mass neutral particles (bubbles) that interact with surrounding water molecules through time-dependent Lennard-Jones potentials [104]. However, the transient high-temperature state produced by cavitation bubbles during the contraction and collapse phase is often not considered, and the instantaneous high temperature may reach as high as 3000 K [105].

Electric and magnetic fields are also commonly used in the non-thermal processing of food. Perhaps in the future, it can be used for the mining of bioactive peptides, such as assisting enzymatic hydrolysis and promoting the active conformation of polypeptides. Magnetic field simulations currently are implemented in DL_POLY_4 [106].

## 7. Artificial Intelligence Promotes Virtual Screening of Bioactive Peptides

### 7.1. Simulation and Algorithm Improvement

AI, particularly machine learning, has been employed to develop predictive force fields, enhancing the accuracy of molecular dynamics simulations [107]. AI is trained on datasets generated by quantum mechanics calculations and uses adaptive algorithms to adjust simulation parameters dynamically. This allows for more accurate simulations of complex biological processes. In ab initio molecular dynamics simulations, machine learning interatomic potentials (MLIPs) have been developed, achieving more efficient accuracy. Meanwhile, Cui et al. [108] applied a dual-layer self-supervised learning scheme to MLIPs, extracting structural information from global and local perspectives, further reducing prediction errors and simulation instability. Another study developed an AI-based ab initio biomolecular dynamic system (AI2BMD), which uses protein fragments and machine learning force fields to achieve efficient and precise simulations of full-atom large molecules. Compared to density functional theory, it reduces computational time by several orders of magnitude. The system demonstrates the ability to explore the dynamic changes in the conformational space of active peptides, compensating for dynamic changes at the atomic level over time that cannot be observed in wet-lab experiments [109].

Furthermore, deep learning has also shown excellent performance in mass spectrometry-based proteomics. Deep learning models can predict peptide retention times, ion mobility, collision cross-sections, and other mass spectrometry properties from amino acid sequences alone [110]. This compensates for a range of shortcomings in peptide identification by mass spectrometry, allowing peptidomics to develop further. In the future, the combination of quantum computers and AI will further advance the simulation of biomolecular dynamics.

Even though molecular docking and molecular dynamics have reduced material consumption in wet experiments, this technique still requires a significant amount of computational cost and running time. High-quality small datasets for the establishment of predictive models and predicting the properties of unknown peptides at a higher order of magnitude may be a more effective and cost-effective comprehensive approach [111].

Predictive models have become powerful tools for exploring complex biological issues. However, these powerful models are also a “black box”. It is unclear whether researchers truly understand the basis for the decisions made by the models, as well as how the input features change to become decision outputs. To address this challenge, tools such as SchNet4AIM have been developed to interpret the decision-making behavior of complex models [112].

### 7.2. Protein Structure Prediction

In molecular dynamics, studying various protein changes at the molecular level first requires constructing a credible and realistic protein spatial structure model. However, the number and variety of food-related protein crystal structures are limited, which restricts the application of molecular dynamics simulations in food protein research. Unlike X-ray, nuclear magnetic resonance, or cryo-electron microscopy, computer-predicted protein structures only require the protein’s amino acid sequence, greatly reducing the difficulty of obtaining protein three-dimensional structures. Computer prediction of the correct folding of amino acid sequences into protein structures is complex. Currently, computer prediction of protein structures is mainly divided into three methods: homology modeling, de novo prediction, and deep learning prediction models [113,114].

Homology modeling involves obtaining the target protein’s amino acid sequence from protein databases (such as UniProt, PDB, etc.) and using algorithms like BLAST (https://blast.ncbi.nlm.nih.gov/Blast.cgi accessed on 5 August 2025, Basic Local Alignment Search Tool) to search for known structured proteins with high homology to the target sequence. Sequence alignment tools such as Clustal Omega and MAFFT are used to calculate similarity scores, such as expectation values and alignment coverage, to assess the similarity between sequences. Suppose the homology between the target and template sequences is high enough (usually greater than 30%): in that case, homology modeling software (such as SWISS-MODEL, MODELLER, etc.) can be used for structure prediction [115].

De novo prediction methods are used for protein structure prediction for proteins lacking known structural templates, such as Rosetta@home and I-TASSER. These methods do not rely on known structures but instead use the physicochemical properties of amino acids, such as hydrophobicity, size, and charge, to predict their tendencies in protein structures. They also utilize statistical potential energy functions, such as Rosetta’s knowledge-based potentials, which are based on statistical analyses of many known structures to assess the reasonableness of protein conformations. Additionally, Monte Carlo simulations randomly explore the protein conformational space in search of low-energy stable structures. Further optimization is achieved through genetic algorithms that simulate natural selection processes. Finally, molecular dynamics (MD) simulations are used to study the dynamic behavior of proteins at the atomic level to predict their structures. De novo prediction is a complex process whose accuracy is affected by various factors, including algorithm efficiency, the accuracy of potential energy functions, and the ability to explore conformational space [113]. Although de novo prediction provides valuable structural information in some cases, its high computational cost and relatively low accuracy often limit its use as an auxiliary method combined with other structural prediction techniques. With the improvement of computing power and algorithms, the accuracy and application range of de novo prediction have been further enhanced [108].

Deep learning models are regarded as an independent prediction method in protein structure prediction, differing from traditional homology modeling and de novo prediction but incorporating features of both. Deep learning models rely on a large amount of known protein structure data for training, similar to how homology modeling depends on known structural templates. Deep learning models directly learn the mapping from amino acid sequences to three-dimensional structures without complex intermediate steps, aligning with the goal of de novo prediction [116]. Deep learning models simultaneously predict proteins’ secondary, tertiary, and even quaternary structures, while homology modeling and de novo prediction typically focus on specific structural levels. Deep learning models use attention mechanisms to capture long-range dependencies between amino acid sequences, aiding in understanding remote interactions in protein structures. Unlike other homology modeling methods, deep learning models do not require known structural templates for structure prediction. They predict protein structures from scratch (de novo). The latest deep learning models, such as AlphaFold3, predict protein structures and the structures and interactions of other biomolecules like DNA, RNA, and ligands. AlphaFold3 has been assessed on the PoseBusters benchmark set for protein–ligand interface performance, with prediction accuracy exceeding that of docking tools Vina and Gold [114]. However, deep learning models require substantial computational resources during the training phase; once trained, they can perform structure predictions quickly, which is valuable for large-scale proteomics studies [116]. Deep learning models have good generalization capabilities and predict various protein structures, including those that are difficult to determine using traditional methods.

### 7.3. Mining of Active Peptides

AI is fundamentally transforming the paradigm for discovering bioactive peptides, with its core breakthrough lying in the enhanced ability for cross-scale functional prediction. Past extensive wet-lab experiments have amassed vast, high-quality datasets. These provide the foundation for AI to mine data features and build highly accurate predictive models. Traditional bioactive peptide screening relied on trial-and-error experiments, whereas AI integrates multidimensional biological information (sequence, structure, dynamic interactions) to achieve precise functional prediction and targeted peptide design.

In the field of antimicrobial peptide (AMP) prediction, AMPs must simultaneously satisfy multiple properties: antimicrobial activity, low cytotoxicity, and high stability. For example, EvoGradient combines iterative gradient descent with projection operations to perform in silico directed evolution of peptide sequences. By analyzing peptide sequences encoded in the genomes of clinically isolated multidrug-resistant pathogens, peptides like pep-19-mod were identified. This peptide effectively cleared clinically isolated vancomycin-resistant enterococci in a mouse thigh infection model upon intraperitoneal or localized intramuscular injection, with no significant toxicity observed [117]. The sAMPpred-GAT model utilizes predicted peptide structural information to identify peptides. Graph attention network (GAT) analysis is then performed on the graphs to learn the discriminative features. Experimental results show that sAMPpred-GAT outperforms the other state-of-the-art methods in terms of AUC [118].

The function of self-assembling peptides (SAPs) is highly dependent on structural order. Deep generative models enabling sequence–structure–function interlocked design are driving material innovation. The TransSAFP model employs transfer learning to establish a quantifiable relationship between β-sheet content and antimicrobial/mechanical properties. The self-assembled peptide precursor peptide designed by this model demonstrated excellent therapeutic effects against intestinal bacterial infections in mice. In addition, it demonstrates an enhanced biofilm eradication ability and does not induce acquired drug resistance [119].

In conclusion, AI-driven functional peptide prediction has formed a closed-loop “data-algorithm-validation-translation” framework. By decoding sequence patterns, dynamic interactions, and material functions, AI empowers the efficient development of therapeutic peptides and SAPs. This provides transformative tools to address the antimicrobial resistance crisis and advance sustainable development.

## 8. Comparison Between Molecular Simulation and Traditional Method of Bioactive Peptide Mining

### 8.1. Drawback of Traditional Mining Methods of Bioactive Peptides

In the past, the primary method for decoding protein libraries to obtain bioactive peptide fragments was hydrolyzing the target protein using various proteases and separating the complex hydrolysate through chromatography columns or preparative liquid chromatography. Subsequently, the activity of different fractions was assessed. Once the fraction with the highest activity was identified, the peptide sequences within that fraction were determined. Finally, the peptides were synthesized for subsequent activity validation.

This technical workflow may not be suitable for obtaining bioactive peptides through protein hydrolysis. However, it remains a favored approach for mining bioactive peptides in processed foods, such as exploring active peptides in ham [120]. This is attributed to the fact that complex food systems cannot yet be emulated on computers. For molecular simulation, the task of mining active peptides in processed foods is more challenging, given the greater diversity of proteins and proteases that contribute to the formation of active peptides in these foods.

#### 8.1.1. Types of Enzymes

**Figure 5:**
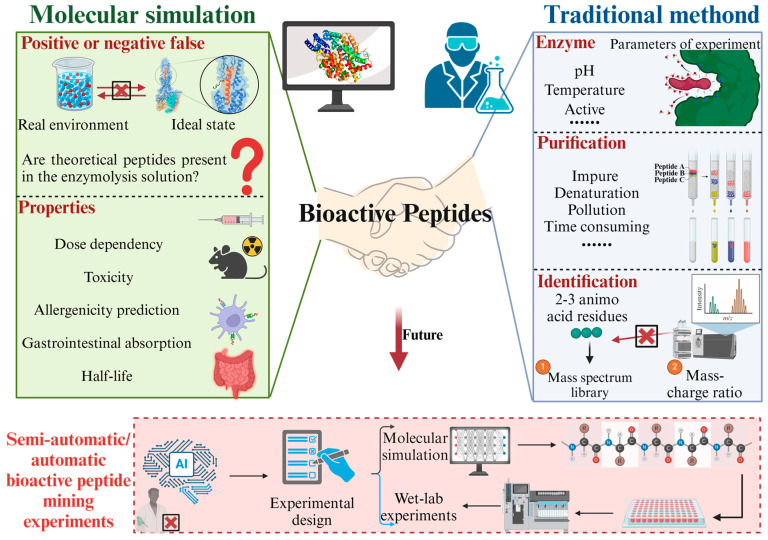
Comparison between molecular simulation and traditional method of bioactive peptide mining. Figure made by BioRender (agreement number: BU28ICEZO6).

Traditional enzymatic hydrolysis for mining bioactive peptides relies on proteinases (Figure 5). Factors such as pH and temperature affect the activity of proteinases and the hydrolysis effect, which may lead to incomplete coverage of hydrolysis sites and insufficient optimization of conditions. This will ultimately result in an incomplete set of bioactive peptides obtained. In molecular simulation, it is possible to study simultaneously the virtual hydrolysis of at least six or more common proteinases in the food industry [36]. This is undoubtedly a more efficient way to screen for bioactive peptides.

#### 8.1.2. Purification of Protein and Bioactive Peptides

The separation and purification of proteins is a key step in obtaining bioactive peptides using traditional methods. Impure proteins lead to incorrect assignment of bioactive peptides, especially when the protein’s peptide sequence has not been fully elucidated. Of course, focusing on predictions of the protein structure in 2D can be highly informative as well (secondary structure, solvent accessibility, etc.) [121]. Based on secondary structure in combination with physicochemical features, peptides can be characterized (in, for example, amphiphilic, transmembrane, hydrophobicity, etc.), see, for example, Gautier et al. [122] and Keller, R. C. A. [123]. The number of protein sequences humans have analyzed is currently around 250 million (including reviewed and unreviewed), which accounts for less than 0.1% of all proteins on Earth (data from the UniProtKB database). The data available for analyzing proteins in food is even more limited.

Traditional bioactive peptide separation methods, such as chromatography columns and preparative liquid chromatography, although capable of separating complex hydrolysates, have limited separation efficiency and resolution. It is difficult to separate all peptide fragments, resulting in some active peptide fragments being mixed and complex to identify further. The separation and purification process usually requires a lot of time and labor, and the operation is complex, which is prone to human error and affects the separation effect. In addition, peptide loss and denaturation during the separation process are common problems, further reducing the recovery rate of active peptides [124]. The active peptides obtained by the molecular simulation technique are verified by solid phase synthesis of peptides, which avoids the problem of rich concentration of peptide separation [57].

#### 8.1.3. Limitations of Mass Spectrometry

The limitations of mass spectrometry (MS) hardware and software restrict the development of peptidomics, ultimately leading to an incomplete exploration of traditional active peptide mining. MS is not effective in detecting very small and very large peptides. Mass spectrometry has disadvantages for monitoring short peptides (<500 Da, with 2–3 amino acid residues). The limited number of fragment ions and the potential for overlapping *m*/*z* values lead to ambiguous or incorrect sequence assignments. The limited short peptide databases and interference from contaminants further increase the difficulty of separating short peptides from the noise [125]. However, peptides (2–4 amino acid residues) have superior biocompatibility, low immunogenicity, and the ability to self-assemble, and these excellent properties cannot be ignored [126,127]. Recently, the interpretation of mass spectrometry data has been dramatically enhanced by machine learning and deep learning algorithms, leading to significant progress in data interpretation and hypothesis generation in proteomics [128]. In the future, protein sequence analysis will make more substantial progress, which may better serve the traditional methods for screening bioactive peptides.

Otherwise, traditional methods mainly rely on limited wet-lab data, which is insufficient to support the establishment of predictive models based on large datasets. This leads to the weak generalization ability of bioactive peptide data obtained through traditional methods, as they cannot effectively learn the structure–activity relationship of peptides.

### 8.2. Drawback of Computer Mining Methods of Bioactive Peptides

Even though computer hardware has made significant advancements, the current computational power still does not support the simulation of complex food systems. In other words, the computational cost of simulation is not balanced with its application value. At the same time, the relevant force field parameters and algorithms are imperfect, which may lead to calculation errors in such complex food systems. All these limiting factors result in the current situation where the virtual enzymatic digestion and peptide activity exploration of bioactive peptides are still idealized. The research focuses on the perfect enzymatic digestion of a single protein or the ideal binding of a single peptide to a single protein receptor without considering the effects of molecular crowding and enzymatic reaction conditions in real systems. As a result, the current molecular simulation mining of food-derived bioactive peptides has a number of shortcomings.

#### 8.2.1. False Positives or Negatives

In theory, the amino acid sequence fragments obtained through virtual enzymatic digestion are assumed to result from complete hydrolysis of the protein. Due to environmental variables, enzyme activity, molecular crowding, and other factors, protein hydrolysis is often incomplete. This leads to false-positive results in virtual enzymatic digestion. Theoretically obtainable active peptides from virtual enzymatic digestion may not be obtained in actual enzymatic experiments. This requires researchers to adjust enzymatic hydrolysis parameters to obtain the target product continuously. Some of the latest reports have innovated enzymatic hydrolysis methods, such as using batch-coupled hydrolysis technology to improve enzymatic hydrolysis efficiency [129]. The false-positive problem also appears in the subsequent molecular docking or molecular dynamics simulation studies of peptides and target-targeting receptors, so verifying the results of wet-lab experiments is necessary.

Also, false-negative results may occur due to an incomplete understanding of the hydrolytic specificity of enzymes. Therefore, virtual enzymatic digestion is typically used as an initial screening step, with subsequent validation through wet-lab experiments to identify peptide sequences or solid-phase synthesis of peptides for further verification [2,72]. Solid-phase peptide synthesis addresses the acquisition of target peptides required for research, but it may not be suitable for industrial food production. Finally, detecting the molecular simulation of the active peptide in the enzymolysis solution is necessary for its further application in food processing to develop new functional foods. Researchers have combined large language models with tools such as internet search, code execution, and experiment automation to enable this AI system (Coscientist) to independently design, plan, and execute complex experiments, and have been verified in palladium-catalyzed cross-coupled response optimization experiments [130]. For the field of active peptide mining, such AI systems may also be invoked to promote the research of molecular simulation mining of active peptides to ensure their effectiveness, interpretability, and preparability.

#### 8.2.2. Limitations in the Study of Bioactive Peptide Properties

In addition to exploring the binding ability of the peptide to the target receptor to explain its functional activity, the peptide properties such as dose, toxicology, solubility, half-life, and intestinal absorption rate also affect the functional activity of the peptide. In the context of bioactive peptides, dose dependency describes how the biological activity of a peptide changes with its concentration. While molecular simulations provide valuable insights into the interactions of peptides with their targets, they have limitations in accurately predicting dose-dependency due to factors such as the availability of 3D structures, scoring function limitations, flexibility issues, dynamic and unpredictable factors, and validation challenges [131]. For example, in the field of AMPs the use of computational screening methods and subsequent experimental testing is hampered by the lack of standardized design and experimental methods [132]. In recent years, some researchers have established predictive models using high-quality databases and machine learning algorithms to provide predictions of peptide properties, including toxicity and allergenicity prediction (ToxinPred), gastrointestinal absorption (SwissAMDE), and half-life (Plifepred). In the future, it may be possible that all the properties of peptides could be predicted by algorithms.

## 9. Conclusions and Future Perspectives

In conclusion, to overcome the limitations of both molecular simulation and conventional peptide mining, the integration of advanced molecular simulations (including multi-algorithm fusion AI models for virtual screening and dynamics, physics-based simulations of novel preparation techniques like ultrasound/electric fields, and improved analysis of peptide sequences and receptor structures) with targeted wet-lab validation presents a highly promising path forward. Prioritizing the development of interpretable AI models (to mitigate the black box effect), expanding the simulation of non-thermal preparation processes, and refining tools for hydrolytic enzyme discovery and peptide-receptor interaction analysis are critical next steps. Success in these areas will significantly accelerate the discovery progress, enabling more efficient development of bioactive peptide-enriched functional foods.

## Figures and Tables

**Table 1:** Crystal structure information of some key receptor proteins.

Receptor Name	PDB ID	Method and Resolution	Small Molecular Ligands
Keap 1	5GIT	X-RAY DIFFRACTION 2.19 Å	XXT (C_19_ H_26_ O_7_)
	2FLU	X-RAY DIFFRACTION 1.50 Å	Nrf2 (16 amino acid residues)
TLR4	2Z63	X-RAY DIFFRACTION 2.00 Å	Two oligosaccharides
	3VQ2	X-RAY DIFFRACTION 2.48 Å	One oligosaccharide, LP5 (C_34_ H_66_ N O_12_ P), LP4 (C_34_ H_66_ N O_12_ P), MYR (C_14_ H_28_ O_2_), NAG (C_8_ H_15_ N O_6_), DAO (C_12_ H_24_ O_2_)
5-HT	7VOE (5-HT2A)	X-RAY DIFFRACTION 2.90 Å	9SC (Subject of Investigation/LOI, C_23_ H_27_ Cl_2_ N_3_ O_2_), CLR (C_27_ H_46_ O), OLC (C_21_ H_40_ O_4_), 1PE (C_10_ H_22_ O_6_), MG (Mg)
	5TVN (5-HT2B)	X-RAY DIFFRACTION 2.90 Å	CLR (C_27_ H_46_ O), OLC (C_21_ H_40_ O_4_), 7LD (C_20_ H_25_ N_3_ O), PEG (C_4_ H_10_ O_3_), PO4 (O_4_ P)
ACE	1O8A	X-RAY DIFFRACTION 2.00 Å	NAG (C_8_ H_15_ N O_6_), NXA (C_4_ H_7_ N O_4_), ZN (Zn), ACT (C_2_ H_3_ O_2_), CL (Cl)
	6F9T	X-RAY DIFFRACTION 1.60 Å	Two oligosaccharides, D0Z (C_26_ H_40_ N_4_ O_9_ S), PEG (C_4_ H_10_ O_3_), PGE (C_6_ H_14_ O_4_), IMD (C_3_ H_5_ N_2_), ZN (Zn), EDO (C_2_ H_6_ O_2_), BO3 (B H_3_ O_3_), CL (Cl)
Tyrosinase	2Y9X	X-RAY DIFFRACTION 2.78 Å	HO (Ho), 0TR (C_7_ H_6_ O_2_), CU (Cu)
	1WX5	X-RAY DIFFRACTION 2.02 Å	CL (Cl), NA (Na)
XO	1FIQ	X-RAY DIFFRACTION 2.50 Å	FAD (C_27_ H_33_ N_9_ O_15_ P_2_), MTE (C_10_ H_14_ N_5_ O_6_ P S_2_), FES (Fe_2_ S_2_), MOS (H Mo O_2_ S), TEI (C_16_ H_16_ N_2_ O_3_ S)
	1N5X	X-RAY DIFFRACTION 2.80 Å	FAD (C_27_ H_33_ N_9_ O_15_ P_2_), MTE (C_10_ H_14_ N_5_ O_6_ P S_2_), FES (Fe_2_ S_2_), MOS (H Mo O_2_ S), PM6 (C_5_ H_4_ N_4_ S)
	3NVY	X-RAY DIFFRACTION 2.00 Å	FAD (C_27_ H_33_ N_9_ O_15_ P_2_), MTE (C_10_ H_14_ N_5_ O_6_ P S_2_), QUE (C_15_ H_10_ O_7_), FES (Fe_2_ S_2_), MOS (H Mo O_2_ S)
Monoamine oxidase A	2Z5X	X-RAY DIFFRACTION 2.20 Å	FAD (C_27_ H_33_ N_9_ O_15_ P_2_), DCX (C_12_ H_27_ O P), HRM (C_13_ H_12_ N_2_ O), GOL (C_3_ H_8_ O_3_)
	2BXR	X-RAY DIFFRACTION 3.00 Å	FAD (C_27_ H_33_ N_9_ O_15_ P_2_), MLG (C_13_ H_15_ Cl_2_ N O)
Calcium-sensing receptor	7DD7	ELECTRON MICROSCOPY 3.20 Å	H43 (C_24_ H_26_ N_2_ O_2_), NAG (C_8_ H_15_ N O_6_), TRP (C_11_ H_12_ N_2_ O_2_), CA (Ca), CL (Cl)

**Table 2:** Advantages/disadvantages of computational method.

Types	Category	Advantages	Disadvantages
Virtual enzymolysis	Enzyme	Obtain all the peptide sequences that can be theoretically generated.	Enzymatic hydrolysis conditions, such as temperature, pH, and ionic strength, were not fully considered.
Molecular dock	Rigid docking	Ultra-fast computation: ignores ligand-receptor conformational changes; high-throughput screening: handles > 100,000 compounds/day.	Low accuracy: fails to simulate induced-fit effects (binding pose error > 5 Å); limited applicability: only suitable for rigid binding pockets.
	Semi-flexible docking	Balanced precision/efficiency: allows ligand flexibility; broad applicability: simulates small molecules/peptides binding to proteins.	Ignores receptor flexibility: critical residue motions; misses long-range interactions: cannot model electrostatic shielding in membrane proteins.
	Flexible docking	High-precision binding: full conformational sampling (RMSD ≤ 1.0 Å); captures allostery: simulates conformational shifts.	High computational cost: hours-to-days per task; inadequate sampling: prone to local minima; requires enhanced sampling.
Molecular dynamic simulation	All-atom MD	Atomic resolution: resolves hydrogen bonds, water-mediated interactions; biophysical accuracy: simulates enzyme catalysis, ion channel gating.	Extreme computational demand; force field limitations.
	Coarse-grained MD	1000× faster: 4–8 atoms grouped per bead (e.g., Martini force field); simulates ms events; macroscale phenomena: captures membrane self-assembly, protein folding.	Loss of atomic detail: side-chain interactions (e.g., π-π stacking) unquantifiable; poor parameter transfer: system-specific re-parameterization needed.
Deep learning/machine learning models	Various functional prediction models	Considerable accuracy, fast prediction rate and lower prediction cost.	A large number of high-quality databases are needed for training.
	Virtual cell model	Multi-scale integration capability, visual analysis of complex cell relationships, assessment of controllability and traceability.	Data quality dependence, the contradiction between calculation accuracy and efficiency, and the complexity of verification experiments.

## Data Availability

No new data were created or analyzed in this study. Data sharing is not applicable to this article.
